# Life-Threatening Retropharyngeal Hemorrhage Secondary to Rupture of the Inferior Thyroid Artery

**DOI:** 10.1155/2015/789076

**Published:** 2015-12-24

**Authors:** Cristina G. Calogero, Andrew C. Miller, Marna Rayl Greenberg

**Affiliations:** Department of Emergency Medicine, Lehigh Valley Hospital and Health Network, USF MCOM, CC & I-78, Allentown, PA 18103, USA

## Abstract

Inferior thyroid artery (ITA) rupture is rare and may progress to life-threatening conditions. We present a patient who visited the emergency department after an episode of syncope and dizziness in which he had a mechanical fall that resulted in abrasions and a hematoma to his left forehead. The patient presented with dysphagia and anterior neck swelling that progressed rapidly into airway compromise requiring endotracheal intubation. Emergent computed tomography revealed a large retropharyngeal hematoma, with active arterial extravasation that was thought to be arising from the thyrocervical trunk on the left. The hematoma measured approximately 6.7 cm transversely and 3.2 cm anteroposteriorly and extended from the level of the lower nasopharynx, down the neck into the retropharyngeal and danger space and into the mediastinum posterior to the esophagus, overall approximately 25 cm. The larynx was deviated anteriorly and there was esophageal compression. An emergent arteriogram and catheterization confirmed bleeding from branches of the ITA, and successful embolization was performed. It is important to recognize the ITA rupture as a potential etiology of an acute airway compromise. In emergent situations, while securing an airway is a priority, rapidly initiating diagnostic testing to confirm the diagnosis and arranging for arterial embolization can be life-saving.

## 1. Introduction

Aneurysm and rupture of the inferior thyroid artery (ITA) are rare and less than 25 cases have been reported in the literature, the first reported in 1959 [1–5]. Rupture of the ITA is a rare cause of mediastinal hemorrhage, with less than 70 cases reported since 1966 [6–9]. A retropharyngeal hematoma large enough to cause life-threatening conditions can develop hours or days after a precipitating injury in which the patient may initially present with hoarseness and mild dysphagia and progress to severe respiratory distress [7, 9]. Due to the anatomic location, a hematoma large enough can occlude the airway at the level of the pharynx and compress surrounding structures causing life-threatening circumstances [1, 7]. We report a unique case in which a retropharyngeal hematoma occurred secondary to a ruptured ITA resulting in airway compromise that required endotracheal intubation. ITA ruptures and retropharyngeal hematomas need to be quickly identified and managed in order to prevent a potentially lethal outcome [1, 8].

## 2. Case Report

An 80-year-old male presented to the emergency department (ED) after an episode of syncope and dizziness in which he awoke on the floor earlier that morning. The patient did not complain of a headache or neck pain but expressed concern with dysphagia. The patient had a cardiac history consisting of controlled response ventricular atrial fibrillation and was removed from his Coumadin two weeks priorly on the recommendation of his cardiologist due to recurrent dizziness, syncope, and frequent falls. His records revealed that his cardiologist felt his history of dizzy and syncopal episodes was not due to a cardiac etiology. He was on sotalol for the atrial fibrillation. Two years priorly the patient had two-vessel coronary artery bypass surgery. Additionally he had a history of hyperlipidemia and gastroesophageal reflux disease and was due for surgery on a 4.8 cm abdominal aortic aneurysm.

On examination, his vital signs were temperature 97.7°F, blood pressure 160/94 mmHg, pulse rate 65 beats/minute, respiratory rate 18 breaths/minute, and oxygen saturation 100%. He had a small abrasion and contusion on his left forehead. He was awake, alert, and coherent but appeared uncomfortable with prominent soft tissue in the anterior neck. His heart rate had a controlled ventricular response and his lungs were clear to auscultation. His coagulation laboratory studies were normal.

Very rapidly over the next ten-fifteen minutes the patient had more difficulty swallowing and obvious swelling started evolving in the anterior neck region on the left. Within thirty minutes his dysphagia progressed and the patient visibly had increasing fullness on the left side of his neck and anterior chest wall without crepitus. The patient became hoarse and began to posture into the sniff position. Due to airway compromise, endotracheal intubation with mechanical ventilation was performed without complication. An enhanced computed tomography (CT) of the soft tissues of the neck, cervical spine, and abdomen was performed. The chest CT revealed a large retropharyngeal hematoma posterior to the level of the thyroid gland with active arterial extravasation thought to be coming from the thyrocervical trunk. The hematoma measured 6.7 cm transversely and 3.2 cm anteroposteriorly and extended from the level of the lower nasopharynx down the neck into the retropharyngeal space and into the mediastinum posterior to the esophagus, totaling 25 cm. The larynx was deviated anteriorly by the hematoma, in addition to esophageal compression. The cervical spine CT revealed prevertebral soft tissue swelling displacing the endotracheal tube anteriorly. The CT of the abdomen incidentally confirmed the 4.8 cm abdominal aortic aneurysm.

An emergency arteriogram and embolization were requested. Selective catheterization of the left thyrocervical trunk revealed evidence of a large hemorrhage coming from branches of the ITA which was subsequently successfully embolized.

## 3. Discussion

Patients with an ITA rupture can either be asymptomatic or present with symptoms such as hoarseness, dysphagia, or swelling in the neck [2–4, 6]. The differential is complex on these patients because there is overlap in some of the symptoms with those of an allergic reaction, a stroke (unilateral presentation), or infectious etiology. In our case, it seemed more than coincidental that the patient experienced a mechanical fall priorly, and it likely caused the ITA lesion and eventual retropharyngeal hematoma. The swelling compressed the esophagus and trachea, deviated the larynx, and displaced the endotracheal tube anteriorly, compromising the airway. ITA ruptures follow an acute and potentially fatal course.

Emergency medicine physicians are frequently the first point of contact for such symptoms, so correct diagnosis and management are essential in preventing fatal outcomes. In emergent situations, a secure airway needs to be established, an enhanced CT and angiography allow for diagnosis, and arterial embolization is minimally invasive and should be the first choice for stopping the bleeding [1–3, 6, 8–10].
